# Successful Treatment of Spontaneous Cerebrospinal Fluid Rhinorrhea With Endoscopic Third Ventriculostomy and Lumboperitoneal Shunt: A Case Report

**DOI:** 10.3389/fnins.2020.00057

**Published:** 2020-01-31

**Authors:** Chao Tang, Junhao Zhu, Kaiyang Feng, Jin Yang, Zixiang Cong, Xiangming Cai, Liang Qiao, Chiyuan Ma

**Affiliations:** ^1^Department of Neurosurgery, Jinling Hospital, School of Medicine, Nanjing University, Nanjing, China; ^2^School of Medicine, Nanjing Medical University, Nanjing, China; ^3^Arkansas Colleges of Health Education, Fort Smith, AR, United States; ^4^School of Medicine, Southeast University, Nanjing, China

**Keywords:** spontaneous CSF rhinorrhea, ETV, LPS, treatment, case report

## Abstract

Spontaneous cerebrospinal fluid (CSF) rhinorrhea represents an important clinical entity that is being observed with increasing prevalence, ranging from 14 to 55%. Spontaneous CSF rhinorrhea is associated with elevated intracranial pressure (ICP), which is rarely stopped without surgical intervention. Endoscopic endonasal repair is typically warranted for CSF rhinorrhea. However, the recurrence rate of CSF leaks after the endoscopic endonasal repair of skull base defects due to ICP is usually high. We describe a 25-year-old man without a history of head injury, tumor, or obesity. The onset of his symptoms occurred in 1 week in the form of a persistent clear left nostril rhinorrhea. Computed tomography (CT) and magnetic resonance images (MRI) showed signs of CSF in the left sphenoidal sinus, meningocele in the left frontal sinus, empty sella, hydrocephalus, and Chiari I malformation (CIM). Cine-MRI revealed the flow of CSF was obstructed at the aqueduct and the outlet of the fourth ventricle. Endoscopic third ventriculostomy (ETV) was performed for the patient with obstructive hydrocephalus. Post-operative CSF pressure measurement demonstrated elevated ICP. The patient still had case of CSF rhinorrhea, and subsequently underwent lumboperitoneal shunt (LPS) for treatment of ICP. The patient showed a prompt resolution of CSF leak. Ten months later, the patient showed a significant improvement in terms of his herniated tonsil and cessation of CSF rhinorrhea.

## Introduction

Spontaneous cerebrospinal fluid (CSF) rhinorrhea occurs in the absence of traumatic, tumor, or iatrogenic disorder, which usually makes up about 3–4% of CSF leaks (Lopatin et al., 2003; Shah et al., 2017). Over the last decade, significant attention has been given to the role of increased intracranial pressure (ICP) as a contributing factor for spontaneous CSF rhinorrhea (Schlosser et al., 2003; Woodworth et al., 2008; Jindal et al., 2009).

Endoscopic endonasal repair is the preferred surgical intervention for spontaneous CSF leaks, but reported recurrence rates vary widely in the literature, ranging from 25 to 87% (Shetty et al., 2000; Friedman and Jacobson, 2002; Schick et al., 2003; Schlosser and Bolger, 2003a, b; Schlosser et al., 2003, 2006; Martin and Loehrl, 2007; Brazis, 2008; Lee et al., 2011; Harvey et al., 2012). Multiple investigators suggest that control of an elevated ICP can improve success rates of endoscopic repair of patients with spontaneous CSF fistula equivalent to those of other causes (Carrau et al., 2005; Woodworth et al., 2008).

So spontaneous CSF rhinorrhea management requires adjunctive measures to supplement the surgical repair of the leak site including conservative treatment, repeated lumbar punctures, and/or CSF diversion to reduce the ICP (Rosenfeld et al., 2013).

Optimal treatment strategies for spontaneous CSF rhinorrhea are not clearly established. In the present case, we describe the case of a patient who presented with spontaneous CSF rhinorrhea due to an elevated ICP. The patient was managed successfully without repair of the dural defect via endoscopic third ventriculostomy (ETV) and lumboperitoneal shunt (LPS).

## Case Report

The patient was a 25-year-old man with no history of head injury, tumor, or obesity. He presented with complaints of a watery discharge from his left nostril for 1 week. He reported that the nasal discharge was associated with a headache without fever. On physical examination, no other abnormalities were noted.

**FIGURE 1:**
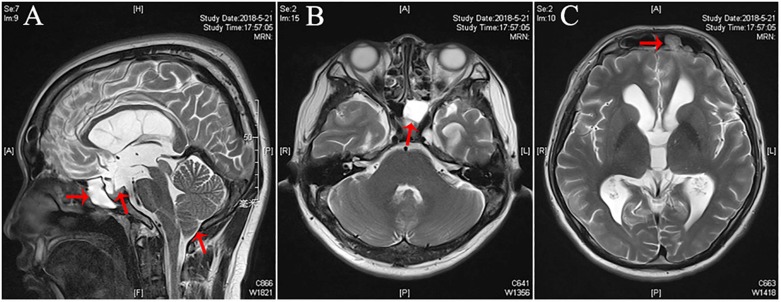
Pre-operative imaging of the patient with CSF rhinorrhea. **(A)** Pre-operative sagittal T2-weighted magnetic resonance imaging (MRI) demonstrating hyperintense in the left sphenoidal sinus, partial empty sella, and Chiari I malformation (CIM) (arrows). **(B)** Pre-operative axial T2-weighted MRI demonstrating hyperintense in the left sphenoidal sinus (arrows). **(C)** Pre-operative axial T2-weighted MRI demonstrating a left sided meningoencephalocele (arrow) extending into the left frontal sinus.

The head preoperative computed tomography (CT) of the patient showed abnormal density in the left frontal sinus and left sphenoidal sinus. Moreover, the brain magnetic resonance images (MRI) demonstrated bilateral enlarged lateral ventricles because the Evans’ index (EI) is greater than 0.3 (EI = 0.42) (Relkin et al., 2005). T2-weighted MRI showed meningocele in the left frontal sinus, hyperintense in the left sphenoidal sinus, partial empty sella, and Chiari I malformation (CIM) (Figure 1). A Cine-MRI revealed that the flow of CSF was obstructed at the aqueduct and the outlet of the fourth ventricle.

## Surgical Approach

**FIGURE 2:**
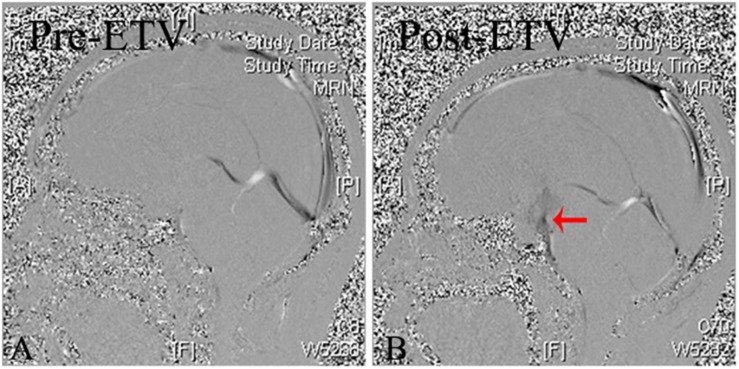
Cine-MRI of pre- and post-ETV. **(A)** Pre-ETV sagittal Cine-MRI demonstrating no CSF flow at aqueduct and the outlet of the fourth ventricle. **(B)** Post-ETV sagittal Cine-MRI demonstrating the moving CSF flow across the bottom of the third ventricle (arrow).

An ETV was performed for reconstructing the CSF circulation (Figure 2). Yet his CSF rhinorrhea remained unresolved after ETV; opening CSF pressure was 33 cm H_2_O by lumbar puncture. A lumbar drain was inserted in immediately post operation to last 1 week in order to reduce CSF pressure. No CSF rhinorrhea was observed during the lumbar drain period. However, it reoccurred afterward. Then LPS was performed to decrease ICP and rhinorrhea cessation on day 1 after shunting.

**FIGURE 3:**
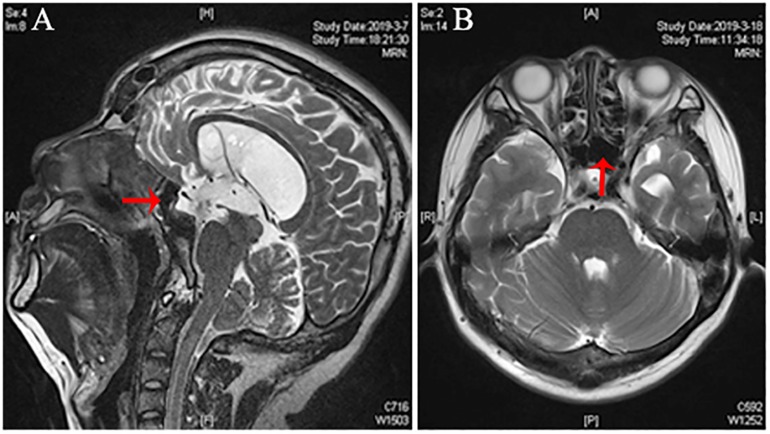
Post-ETV MRI of the patient with CSF rhinorrhea. **(A,B)** Post-ETV sagittal and axial T2-weighted MRI demonstrating CSF flow signal disappeared significantly compared to the preoperative image in the left sphenoidal sinus (arrow).

An MRI at 10 months after the surgery showed improved cerebellar tonsillar hernia, signs of CSF in the left sphenoidal sinus, and that meningocele had also disappeared (Figure 3). The patient has been symptom-free without recurrence of rhinorrhea throughout the 12 months of follow-up period.

## Discussion

Spontaneous CSF rhinorrhea without an identifiable inciting event rarely resolves spontaneously, and if left untreated may increase the risk of ascending bacterial meningitis. The pathologic mechanism of spontaneous CSF leaks is still not well understood. Over the last decade, mounting evidence indicates that elevated ICP could be a contributing factor for spontaneous leaks (Schlosser and Bolger, 2003a; Schlosser et al., 2003; Woodworth et al., 2008).

Although endoscopic repair methods can achieve a more than 90% success rate for CSF leaks as a whole, the historical success rates for spontaneous leaks were much worse (Wise and Schlosser, 2007). Many researchers have shown that CSF pressure elevation following rhinorrhea repair accounts for the recurrence after successful surgical management (Wang et al., 2011; Chaaban et al., 2014). Once the CSF fistula is closed, ICP tends to increase because CSF diversion into the nasal cavity no longer exists. Recent studies suggest that decreased ICP can improve success rates in patients with spontaneous CSF leaks equivalent to those with other causes (Lloyd et al., 2008; Martin-Martin et al., 2012; Bernal-Sprekelsen et al., 2014; Martinez-Capoccioni et al., 2015). Therefore, if the elevated ICP of the patient is not addressed, despite successful surgical repair, patients often have a recurrence at the same site, or at a distant site (Gassner et al., 1999; Wang et al., 2011).

At present, interventions to reduce ICP have been more extensively studied in patients with spontaneous CSF leaks. Previous studies reported that patients are generally obese middle-aged females and may present with elevated ICP and symptoms of CSF leaks. However, our patient was a 25-year-old man without a history of trauma or obesity. Recent studies support the role of reducing ICP via medication, weight loss, or CSF diversion to improve the recurrence rates of spontaneous CSF leaks after endoscopic repair (Seth et al., 2010; Wang et al., 2011; Chaaban et al., 2014). However, we propose that the reduction in CSF pressure alone leads to resolution of CSF leaks.

We reported a case of CIM with progressive hydrocephalus and syringomyelia that was successfully treated by ETV (Wen et al., 2014). ETV is an effective treatment for hydrocephalus with CIM/syringomyelia. In our case, CSF flow was obstructed at the aqueduct and outlet of the fourth ventricle, proved by cine-MRI, which might indicate the existence of a regional occlusion. Moreover, the patient presented with meningocele, and partial empty sella turcica were found on T_2_ images. T_2_ images were hyperintense in the left sphenoidal sinus compared to the brain, showing the signaling characteristics of CSF. Patients with spontaneous CSF leaks commonly have radiologic evidence of elevated ICP, including meningocele formation, ranging from 50 to 100% (Schlosser and Bolger, 2002). The most common sign is an empty or partially empty sella turcica (Schlosser and Bolger, 2003a). These data indicated that the most probable cause of spontaneous CSF leaks in our case is elevated ICP due to obstruction of the aqueduct and outlet of the fourth ventricle.

**FIGURE 4:**
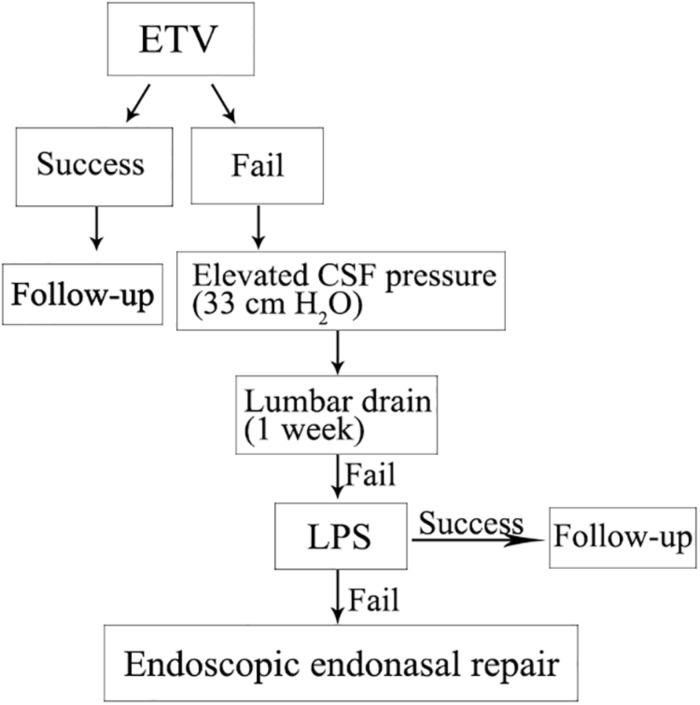
Treatment strategies for the patient with spontaneous CSF rhinorrhea were established according to the protocol shown.

Therefore, in the present case, restoring CSF flow should firstly considered by ETV. Treatment strategies for the patient were established (Figure 4). However, after operation day 2, CSF rhinorrhea still existed despite the third ventricular floor having been opened. The CSF pressure of 33 cm H_2_O was measured by lumbar puncture. Lumbar drains have been used to transiently reduce CSF pressure in patients with high pressure CSF leaks, with increased success rates of CSF fistula repair (Carrau et al., 2005; Woodworth et al., 2008). In the present patient, CSF rhinorrhea was alleviated by lumbar drainage for 1 week. However, CSF rhinorrhea occurred again after lumbar drainage, probably because of the persistently high ICP. It was already reported that a patient with spontaneous CSF rhinorrhea was managed successfully by a VPS alone (Darouassi et al., 2016). Even if the endoscopic repair for the present case had not been carried out yet, placement of a permanent CSF drain, in the form of a VPS or LPS, could be considered in this case. No prospective controlled trials had been made to guide the choice of procedure (Markey et al., 2016). There are a number of advantages to LPS when compared with VPS; LPS avoid intracranial risks such as cerebral hemorrhage, seizures, and shunt malposition (Alkherayf et al., 2015). LPS are also reported to be associated with a lower infection rate than VPS (Aoki, 1990; Choux et al., 1992). First and foremost, LPS was used to avoid accessing ventricular cavities within the brain parenchyma, and to reduce the risk of brain injury, which is why we chose this procedure.

Cessation of the CSF rhinorrhea was observed at a 10 months’ follow-up after shunt surgery. LPS were previously associated with the development of CIM, especially in pediatric patients (Chumas et al., 1993; Kamiryo et al., 2003). However, in the present case, LPS did not lead to any significant increased risk of CIM. Serious over-drainage symptoms were not observed in our case. Even if the patient showed no significant improvements in ventricles within 10 months after the operation compared with pre-ETV, MRI did show improved herniated tonsils.

## Conclusion

The most important factor for successful treatment of spontaneous CSF rhinorrhea and CIM in this patient was reducing ICP through ETV and LPS. A decrease of CSF pressure alone for the treatment could be considered in some cases, as illustrated by our observation. However, further studies are necessary.

## Data Availability Statement

All datasets generated for this study are included in the article/supplementary material.

## Ethics Statement

The studies involving human participants were reviewed and approved by the Ethical Committee of Nanjing Jinling Hospital. The patients/participants provided their written informed consent to participate in this study. Written informed consent has been obtained from the patient for the publication of any potentially identifiable images or data included in this article.

## Author Contributions

CM contributed to the conception of the study. CT performed the investigation and data curation. KF modified the grammar mistakes. JZ, JY, ZC, and XC contributed significantly to the analysis and manuscript preparation. LQ helped to perform the analysis with constructive discussions.

## Conflict of Interest

The authors declare that the research was conducted in the absence of any commercial or financial relationships that could be construed as a potential conflict of interest.
